# Genomic Landscape and Therapeutic Implications of Metaplastic Breast Carcinoma: Insights from a Nationwide Database Including Diagnostic Mimickers

**DOI:** 10.3390/ph19020311

**Published:** 2026-02-12

**Authors:** Shuhei Suzuki, Manabu Seino, Hidenori Sato, Masaaki Kawai, Jiro Ogura, Yuki Hoshi, Yosuke Saito, Koki Saito, Yuta Yamada, Koshi Takahashi, Ryosuke Kumanishi, Tadahisa Fukui, Masanobu Takahashi

**Affiliations:** 1 Department of Clinical Oncology, Yamagata University School of Medicine, 2-2-2 Iida-nishi, Yamagata 990-9585, Japan; 2 Yamagata Hereditary Tumor Research Center, Yamagata University, 1-4-12 Kojirakawa, Yamagata 990-8560, Japan; 3 Obstetrics and Gynecology, Yamagata University School of Medicine, 2-2-2 Iida-nishi, Yamagata 990-9585, Japan; 4 Genomic Information, Yamagata University School of Medicine, 2-2-2 Iida-nishi, Yamagata 990-9585, Japan; 5 Surgery I, Yamagata University School of Medicine, 2-2-2 Iida-nishi, Yamagata 990-9585, Japan; 6 Pharmacology, Yamagata University School of Medicine, 2-2-2 Iida-nishi, Yamagata 990-9585, Japan; 7 Genetic Counseling, Yamagata University School of Medicine, 2-2-2 Iida-nishi, Yamagata 990-9585, Japan

**Keywords:** Metaplastic breast carcinoma, comprehensive genomic profiling, precision oncology, *TP53*, *PIK3CA*, chemotherapy response, C-CAT database, rare breast cancer

## Abstract

**Background/Objectives**: Metaplastic breast carcinoma (MpBC) is a rare and aggressive malignancy characterized by significant histological heterogeneity and limited response to standard chemotherapy. Due to its morphological diversity, MpBC often presents diagnostic challenges and can overlap with other mesenchymal tumors. This study aimed to characterize the genomic landscape of MpBC using a nationwide Japanese database and to explore the molecular basis of its diagnostic ambiguities and therapeutic responses. **Methods**: We retrospectively analyzed genomic and clinical data from 123 MpBC cases registered in the Center for Cancer Genomics and Advanced Therapeutics (C-CAT) database. To evaluate diagnostic boundaries, genomic profiles of histological mimickers, including 19 cases of angiosarcoma and eight cases of myoepithelial carcinoma, were also examined. Furthermore, an exploratory single-cell RNA-sequencing analysis was performed on 3274 cells from independent MpBC datasets to investigate cellular heterogeneity and potential lineage plasticity. **Results**: *TP53* (73.2%) and *PIK3CA* (46.0%) were the most prevalent genomic alterations in the MpBC cohort. Exploratory analysis suggested that *PIK3CA* mutations may be associated with an improved disease control rate in patients receiving taxane-based therapy (*p* = 0.028). Comparisons with mimickers identified distinctive molecular signatures, such as *MED12* and *HRAS* hotspot mutations, across entities. Single-cell transcriptomics identified a distinct subpopulation (7.02% of malignant cells) co-expressing epithelial and phyllodes-like signatures. **Conclusions**: These findings suggest that MpBC harbors hybrid malignant cell populations that may contribute to its complex morphological diversity. While the therapeutic associations are based on a limited cohort and require prospective validation, the integration of comprehensive genomic and single-cell profiling provides an exploratory framework that may potentially enhance diagnostic accuracy in the future. However, these associations remain preliminary and require prospective validation to confirm their clinical utility.

## 1. Introduction

Metaplastic breast carcinoma (MpBC) represents a rare but increasingly recognized subtype of invasive breast cancer, accounting for less than 1% of all newly diagnosed breast malignancies worldwide [1,2]. Yet this uncommon entity has captured growing clinical attention due to its distinctly aggressive behavior and resistance to conventional therapeutic approaches that are effective in other breast cancer subtypes. The World Health Organization defines MpBC as an invasive carcinoma showing differentiation toward squamous cells and/or mesenchymal components, a morphological diversity that reflects the underlying biological complexity of these tumors [3].

The clinical significance of MpBC extends far beyond its rarity. These tumors frequently present with advanced features and are predominantly triple-negative, lacking expression of estrogen receptor, progesterone receptor, and HER2. However, unlike conventional triple-negative breast cancers, MpBC demonstrates a particularly poor response to standard chemotherapy regimens, leading to inferior clinical outcomes [4]. This therapeutic resistance has prompted intense interest in understanding the molecular mechanisms that drive both the unique morphological features and the aggressive clinical behavior of MpBC.

From a diagnostic standpoint, the morphological heterogeneity of MpBC creates substantial challenges [5]. The various histological patterns—including spindle cell, squamous, and matrix-producing variants—can overlap with other rare breast lesions and even non-mammary malignancies. This diagnostic complexity is compounded by inconsistent immunohistochemical marker expression, occasionally leading to misclassification and delayed appropriate treatment. The integration of molecular profiling has begun to provide additional diagnostic insights, revealing genomic alterations that may help distinguish MpBC from morphologically similar entities [6].

Recent advances in comprehensive genomic profiling (CGP) have started to illuminate the molecular landscape of MpBC [7]. Initial studies suggest frequent alterations in tumor suppressor genes and pathways related to epithelial–mesenchymal transition, consistent with the histological plasticity observed in these tumors [8]. However, the rarity of MpBC has historically limited large-scale integrated genomic and clinical analyses. In Japan, the establishment of the Center for Cancer Genomics and Advanced Therapeutics (C-CAT) as a nationwide genomic database has created unprecedented opportunities for studying rare malignancies [9,10]. This comprehensive repository combines detailed genomic profiling with clinical annotations from across the country, offering the potential to overcome the sample size limitations that have constrained previous MpBC research. The systematic collection of both molecular and clinical data provides a unique platform for exploring genotype–phenotype correlations and treatment outcomes in real-world settings [11]. Given the diagnostic challenges, therapeutic resistance, and poor outcomes associated with MpBC, there is an urgent need for improved understanding of its molecular underpinnings. Large-scale genomic analyses may reveal actionable alterations suitable for targeted therapies or identify biomarkers that could guide treatment selection. Furthermore, systematic evaluation of genomic features across a substantial cohort could help clarify the relationship between MpBC and morphologically similar entities, potentially improving diagnostic accuracy.

In this study, we leveraged the comprehensive C-CAT database to characterize the genomic landscape of MpBC within a nationwide Japanese cohort. Our objectives were to define the mutational spectrum of MpBC, explore potential molecular subgroups, and examine associations between genomic alterations and clinical outcomes. By analyzing this large-scale dataset, we aimed to provide insights that could inform precision medicine approaches for this challenging malignancy and potentially improve outcomes for patients with this rare but aggressive form of breast cancer.

## 2. Results

### 2.1. Background

A total of 105,940 cancer patients were registered in the C-CAT database between June 2019 and August 2025. Within this overall cohort, breast cancer was the fourth most prevalent cancer type, comprising 8409 cases and representing 7.9% of the total population. Among these, 123 cases were classified as MpBC based on the OncoTree system and underwent CGP.

Table 1 summarizes the clinicopathological characteristics of the total breast cancer cohort (*n* = 8409). Female sex accounted for more than 99% of patients. The largest age group was patients in their 50s, comprising 2724 individuals (32.3%). Regarding histological subtypes, invasive ductal carcinoma (IDC) was the most frequent, accounting for 5645 cases (67.1%), followed by invasive lobular carcinoma (ILC) with 472 cases (5.6%). The most prevalent genetic alteration was *TP53*, identified in 4455 patients (53.0%). Furthermore, FoundationOne CDx was the most commonly utilized CGP test, performed in 5930 cases (70.5%).

### 2.2. Metaplastic Carcinoma Cases

Table 2 shows the detailed background of the 123 MpBC cases. The median age was 56 years (IQR: 48–66). Current smoking was reported in 17.9% of MpBC patients, and heavy alcohol use in 4.1%. A family history of malignancy was noted in 70.0%. Primary tumor tissue was analyzed in 62.6%, and metastatic samples in 31.7%.

Most MpBC cases demonstrated a triple-negative phenotype. Based on available immunohistochemistry, HER2 expression was detected in only 5.3% of patients, ER in 12.2%, and PgR in 7.3%. Germline *BRCA1* mutation was observed in 0.88% of cases, and no *BRCA2* mutations were identified. These findings confirmed the predominance of the triple-negative phenotype, consistent with prior reports [12].

CGP (Table 3) revealed a complex mutational landscape. The most frequently altered gene was *TP53*, detected in 73.2% of cases. *PIK3CA* mutations were present in 46.0%, followed by *CDKN2A* (33.3%), *PTEN* (26.0%), *CDKN2B* (22.8%), *TERT* (20.3%), and *MYC* (14.6%). Consistent with previous smaller series (PMID 25688406), *TP53* and *PIK3CA* emerged as the most frequently altered genes.

Median tumor mutational burden (TMB) was 3.78 mut/Mb (IQR: 1.26–5.00), consistent with an intermediate mutational burden. Microsatellite instability (MSI) was not detected; one case (0.8%) was reported as equivocal, and all others were microsatellite stable (MSS). Actionable genomic alterations, defined as those for which targeted therapy was available or recommended, were identified in 48.8% of MpBC patients. Cases harbored a *BRAF* V600E mutation [13] and *FGFR2* fusion [14], respectively, representing targetable alterations with approved therapies in Japan for *BRAF* (solid tumor) and *FGFR2* (cholangiocarcinoma only). Among the 123 MpBC cases (Table 4), treatment information was available for 116 patients. Chemotherapy regimens were categorized into four major backbones: taxane-based, anthracycline-based, eribulin-based, and fluoropyrimidine-based therapies. In an exploratory subgroup analysis of taxane-based regimens (*n* = 113), patients with *PIK3CA* alterations achieved a nominally higher disease control rate (DCR) in this exploratory analysis compared with those without (*p* = 0.028). A trend toward better outcomes was observed in *TP53* wild-type tumors, although this did not reach statistical significance (*p* = 0.096). *CDKN2A* and *PTEN* alterations showed no association with improved response to taxane therapy (*p* = 0.591 and 0.602, respectively).

Unlike the patterns observed with anthracycline-based treatment, none of the examined genetic alterations demonstrated significant association with therapeutic response. Specifically, alterations in *TP53*, *PIK3CA*, *CDKN2A*, and *PTEN* showed no predictive value (*p* = 0.787, 0.512, 0.847, and 0.251, respectively). These exploratory findings suggest potential differential associations between genomic alterations and chemotherapy backbone selection for MpBC. Limited data were available for eribulin-based (*n* = 52) and fluoropyrimidine-based (*n* = 56) regimens. In exploratory analyses, no clear genomic predictors of response were identified, likely reflecting small sample sizes and heterogeneous prior treatments.

The differential diagnosis of MpBC encompasses several critical mimickers, including phyllodes tumors [15], angiosarcomas [16], and myoepithelial carcinomas [17]. For example, phyllodes tumors frequently harbor characteristic genetic alterations in *TERT* and *MED12*, which serve as pivotal molecular markers for their diagnosis [18]. In the present cohort, *MED12* alterations were identified in four cases, and an *HRAS* G13R mutation was detected in one case. *HRAS* mutations are recognized as key drivers of myoepithelial differentiation in myoepithelial tumors [19]. The detection of these specific alterations, which are traditionally associated with phyllodes tumors or myoepithelial carcinomas, necessitates rigorous evaluation of whether these cases represent potential misregistration of mimickers, specific lines of differentiation within MpBC, or genuine coexistence of these pathological entities. Distinguishing among these possibilities is crucial for ensuring diagnostic accuracy and understanding the diverse morphological spectrum of MpBC [20] in large-scale genomic databases.

### 2.3. Genomic Landscapes of Histologic Mimickers: Angiosarcoma, Myoepithelial Carcinoma, and Phyllodes Tumors

The diagnostic differentiation of MpBC from its histologic mimickers, such as angiosarcoma, myoepithelial carcinoma, and phyllodes tumors, remains a significant challenge due to overlapping morphological features. To further characterize these rare entities, we analyzed the genomic profiles of 19 angiosarcoma cases and eight myoepithelial carcinoma cases. In the angiosarcoma cohort (*n* = 19), all patients were female with a median age of 49 years. While the clinical database could not definitively distinguish between primary and secondary (e.g., post-radiation) tumors, the majority of cases (89.5%) had received taxane-based chemotherapy. Genomic analysis was performed using various platforms, with FoundationOne CDx being the most frequently utilized (13 cases, 68.4%). The most frequent alterations were *MYC* amplification (68.4%) and *PIK3CA* mutations (42.1%), whereas *TP53* alterations were relatively infrequent (21.1%). Although three cases yielded potentially false-negative results with no clinically significant alterations detected, we identified *HRAS* hotspot mutations (Q61L, *n* = 2; A59T, *n* = 1; G13R, *n* = 1) in a total of four cases, despite the absence of *MED12* or *TERT* promoter variants. In contrast, the myoepithelial carcinoma cohort (*n* = 8) consisted entirely of female patients (median age 55 years); all cases were analyzed using FoundationOne CDx. Notably, *HRAS* mutations were predominant, identified in six cases (Q61K, *n* = 2; Q61R, *n* = 2; Q61H, *n* = 1; G13R, *n* = 1), while the remaining two cases were *HRAS* wild-type. Clinical follow-up showed that while taxanes and anthracyclines were administered in three cases each, the only patient who achieved a therapeutic response was one of the *HRAS* wild-type cases. Furthermore, our previous study of 60 phyllodes tumors [18] identified only a single case with an *HRAS* mutation, which, interestingly, lacked the *MED12* mutations typically seen in that lineage. These genomic findings suggest that while these entities are categorized as distinct diagnoses, there may be a degree of diagnostic overlap or the presence of shared progenitor cells capable of multidirectional differentiation. The identification of *HRAS* mutations across different histologies underscores the complexity of these tumors. To resolve these diagnostic ambiguities and to elucidate the potential coexistence of diverse differentiated cell populations at a higher resolution, we subsequently performed single-cell RNA sequencing.

### 2.4. Single-Cell RNA-Seq Analysis Identifying Hybrid Epithelial-Phyllodes States in MpBC

**Figure 1:**
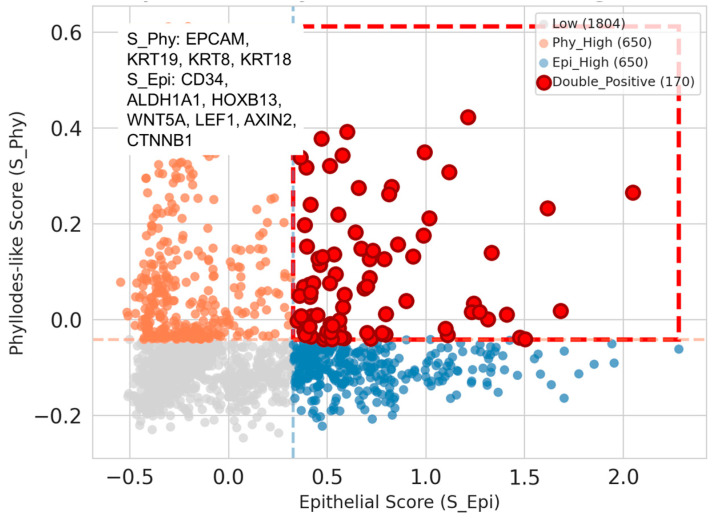
Co-expression of epithelial and phyllodes-like signatures in metaplastic breast carcinoma at the single-cell level. Scatter plot showing the relationship between epithelial signature score (S_Epi, *x*-axis) and phyllodes-like signature score (S_Phy, *y*-axis) in 3274 cells from metaplastic breast carcinoma. Each dot represents a single cell, color-coded by classification: gray (Low, *n* = 1804), blue (Epi_High, *n* = 650), orange (Phy_High, *n* = 650), and red (Double_Positive, *n* = 170). Dashed lines indicate the 75th percentile thresholds (S_Epi: 0.328; S_Phy: −0.041). The double-positive population (upper-right quadrant) represents 5.19% of total cells and 7.02% of malignant cells, with equal distribution across two independent datasets (85 cells each). Pearson correlation analysis showed no significant linear relationship (r = −0.019, *p* = 0.27), indicating independent regulation of the two transcriptional programs.

To generate supportive biological hypotheses regarding the cellular heterogeneity of MpBC at higher resolution, we performed a transcriptomic analysis using the CELLxGENE Census database (version 8 November 2025). We analyzed 3274 cells from two independent datasets (Dataset IDs: de5416ef-bbfa-41f3-99d4-01a3554173f5 and ed880090-465d-4477-9d98-70838ca288e3) [21], of which 2422 were annotated as malignant cells. We evaluated the expression of an epithelial signature (S_Epi: *EPCAM*, *KRT19*, *KRT8*, *KRT18*) [22], representing established luminal epithelial markers, and a phyllodes-like signature (S_Phy: *CD34*, *ALDH1A1*, *HOXB13*, *WNT5A*, *LEF1*, *AXIN2*, *CTNNB1*) [23,24,25,26,27,28], comprising stromal markers and Wnt pathway components associated with phyllodes tumors. Notably, our analysis identified a distinct subpopulation of 170 double-positive malignant cells, representing 7.02% of the malignant cell population (Figure 1). These hybrid cells were reproducible across both independent datasets, each contributing 85 cells to this population. All cells in this double-positive group were confirmed to be malignant. Although representing a minority of cells, the identification of this population across two independent datasets suggests a reproducible biological phenomenon rather than a technical artifact. These data indicate that the morphological overlap between MpBC and phyllodes tumors may reflect the presence of malignant cells with hybrid epithelial–mesenchymal phenotypes, supporting a phyllodes-like conversion hypothesis at the single-cell level.

## 3. Discussion

In this study, we investigated MpBC, a group of breast cancers with limited scientific evidence, utilizing the C-CAT database, one of the largest real-world databases worldwide containing patients’ background and treatment information. We examined patient characteristics, treatment responsiveness, and genetic landscape. Furthermore, given that MpBC presents diagnostic challenges with numerous mimickers and differential diagnoses, we explored the genetic landscape of these entities and attempted partial analysis of associated genes. Our findings confirmed that MpBC exhibits immunohistochemical status similar to triple-negative disease, as previously reported. Taxanes and anthracyclines were employed as the mainstay chemotherapy regimens. However, in current clinical practice, trastuzumab deruxtecan has been approved for HER2-low expression [29] and ultra-low expression (data for ultra-low expression were not available in this study) [30]. Given that approximately one-quarter of cases exhibited low expression, this agent represents a valuable therapeutic option with anticipated efficacy, and further data accumulation is warranted.

Regarding the analysis of genetic profiles and treatment responsiveness, reports on PI3K pathway activation have shown conflicting results, with some demonstrating resistance to taxanes [31] and others showing limited association [32]. In our investigation, a favorable trend in DCR was observed. While these findings should be interpreted with caution due to the limited sample size of the treatment-stratified subgroups, the high frequency of *PIK3CA* alterations—which, along with *TP53*, remains a hallmark oncogenic driver in conventional breast cancer—may potentially suggest a degree of molecular overlap between MpBC and more common breast cancer subtypes. Consequently, these results should be regarded as strictly hypothesis-generating, reflecting the possibility that a subset of MpBC might retain certain therapeutic sensitivities characteristic of conventional breast cancer. This may relatively preserve sensitivity to representative breast cancer chemotherapy regimens. While remaining hypothetical, among these representative chemotherapy agents, the PI3K pathway has been reported to contribute to microtubule stabilization [33], potentially leading to excessive microtubule stabilization by taxanes and subsequent cytotoxicity. Treatment resistance in MpBC remains poorly understood, highlighting the need for further research. Additionally, approximately 10% of tumors demonstrated hormone receptor positivity, suggesting potential benefit from capivasertib [34], with future reports eagerly anticipated. Furthermore, samples harboring representative druggable alterations amenable to relatively tumor-agnostic therapy, such as *BRAF* V600E [13] and *FGFR2* fusion [14], were detected, although approval status varies by country. Given the limited therapeutic options for MpBC, proactive proposal of genomic testing represents an important treatment strategy to maximize therapeutic opportunities for patients.

During our genetic landscape investigation, *MED12* [18,35], a representative genetic signature of phyllodes tumors, and *HRAS* hot spot mutations [19], a characteristic genetic signature of angiosarcoma, were sporadically identified within the MpBC cohort. From a database registration perspective, while possibilities of pathological diagnostic accuracy issues or pull-down selection errors during registration cannot be entirely excluded, we performed exploratory analysis on the possibility that MpBC cells possess genetic aspects of phyllodes tumors and that these may represent diseases with transitional or overlapping spectra [20,36]. The presence of these signature mutations suggests that a subset of MpBC may share common progenitor pathways or exhibit lineage plasticity with its histological mimickers. This molecular commonality likely contributes to the significant diagnostic overlap and morphological diversity observed between these rare breast entities. Analysis of single-cell RNA sequencing data from major public databases revealed the potential existence of a cell population exhibiting both epithelial tumor characteristics and phyllodes tumor features as double-positive. Although this single-cell analysis remains hypothesis-generating for our specific cohort, these preliminary findings underscore the importance of considering the possibility of such cell populations when interpreting large-scale genomic databases. Rather than attributing these observations solely to diagnostic migration or registration errors, future researchers should evaluate whether they represent genuine lineage plasticity within MpBC. Moreover, the possibility that treatment responsiveness may vary depending on these genetic characteristics cannot be excluded, necessitating future prospective validation.

Several limitations exist in this study. Due to the retrospective design, treatment approaches and efficacy assessments were not standardized, and genomic profiling platforms varied among cases. While FoundationOne CDx was predominantly utilized, a small subset underwent testing with the NCC Oncopanel System or Guardant360^®^ CDx, neither of which includes *MED12* in its coverage. This technical heterogeneity potentially leads to an underestimation of certain mutation frequencies when calculated across the entire cohort. These findings highlight the clinical necessity of selecting a genomic testing modality tailored to the differential diagnosis—for example, prioritizing panels that include *MED12* and *TERT* when a phyllodes tumor is suspected—and underscore the importance of future cross-platform standardization in nationwide databases. Furthermore, based on genomic testing indications in Japan (limited to patients who have completed or are expected to complete standard therapy), cases undergoing testing were restricted to those who had completed or were expected to complete standard treatment. Additionally, the small sample size limited statistical power for robust multivariate analysis. Consequently, the absence of multivariable adjustment and multiple testing corrections, combined with real-world data heterogeneity, necessitates that these results be interpreted as hypothesis-generating observations rather than definitive predictive markers. Regarding the pathological assessment, a centralized review of specimens was not conducted due to logistical constraints within the Japanese referral system and the anonymized structure of the database. While registration bias cannot be entirely excluded, the identification of hybrid epithelial-phyllodes populations via single-cell analysis provides an independent biological line of evidence for the observed morphological diversity. Future prospective studies integrating centralized pathology with genomic profiling will be ideal to further validate these findings. Finally, single-cell transcriptome data were obtained from publicly available datasets rather than study-specific samples, making validation of diagnostic accuracy impossible.

This nationwide analysis characterizes the genomic landscape of MpBC and clarifies its relationship with histological mimickers through integrated genomic and single-cell transcriptomic profiling. The integration of morphological and molecular features represents a critical step toward improving diagnostic precision and advancing personalized oncology for MpBC.

## 4. Materials and Methods

### 4.1. Study Design and Data Source

This was a retrospective observational study utilizing the C-CAT nationwide database in Japan. The database includes CG results from patients with advanced solid tumors who underwent molecular testing under the Japanese universal health insurance system. The present analysis used C-CAT database version 20250820, which included data collected between June 2019 and August 2025. Clinical and genomic information was obtained from participating cancer genome-designated hospitals. Approval for this analysis was granted by the C-CAT Data Utilization Review Committee (approval number: CDU2023-032E02) and Institutional Review Board (approval number: 2023-105). The study was conducted in accordance with the ethical guidelines for medical research involving human subjects in Japan. All genomic and clinical data in C-CAT are anonymized and collected with patient consent at the time of CGP testing.

### 4.2. Patient Selection

Patients diagnosed with MpBC were identified in the C-CAT dataset using the OncoTree classification system. All cases classified as MpBC during the study period were included. No additional exclusion criteria were applied, reflecting the exploratory and descriptive nature of the study.

### 4.3. Clinical and Pathological Data

Demographic and clinical characteristics were retrieved, including age at testing, lifestyle factors such as smoking and alcohol consumption, and family history of malignancy. Pathological and sampling-related data were also collected, including specimen type (surgical resection, biopsy, or liquid biopsy), source of specimen (primary versus metastatic site), and available immunohistochemical information. Where available, data on hormone receptor expression and germline status were incorporated.

### 4.4. Genomic Profiling

Genomic profiling was performed using several CGP assays approved for clinical use in Japan during the study period, including the NCC Oncopanel System (Sysmex Corporation, Kobe, Japan), FoundationOne^®^ CDx (Foundation Medicine, Inc., Cambridge, MA, USA), FoundationOne^®^ Liquid CDx (Foundation Medicine, Inc.), Guardant360^®^ CDx (Guardant Health, Inc., Redwood City, CA, USA), and GenMine^®^ TOP Cancer Panel (GenMineLab, Tokyo, Japan). Each platform differs in the number of genes covered and additional features such as assessment of tumor mutational burden (TMB) and microsatellite instability (MSI). The specific platform used for each patient was determined by the treating institution. Genomic data collected included single-nucleotide variants, insertions/deletions, copy number alterations, and gene fusions, as well as global biomarkers such as TMB and MSI status.

### 4.5. Treatment and Outcome Data

Information on systemic therapies administered after genomic profiling was extracted when available. Treatment regimens were broadly categorized (e.g., taxane-based, anthracycline-based, fluoropyrimidine-based, eribulin-based, or other). Treatment outcomes were evaluated by attending physicians according to real-world practice and categorized in the C-CAT dataset as complete response, partial response, stable disease, progressive disease, or not evaluable. For exploratory analyses, DCR was defined as the proportion of patients achieving complete response, partial response, or stable disease.

### 4.6. Single-Cell Transcriptomic Data Processing and Analysis

For the single-cell RNA-sequencing analysis, publicly available data were obtained from the CELLxGENE Census database (version 8 November 2025) using Python (version 3.12) with the cellxgene-census package (version 1.10.0). We queried cells annotated with the disease term “metaplastic breast carcinoma” from the breast tissue category. Raw count data were normalized to a total count of 10,000 per cell and log-transformed. Gene signature scores were calculated using the score_genes function in Scanpy (version 1.9.0), which computes the mean expression of target genes minus the mean expression of control genes with similar expression levels. We defined an epithelial signature (S_Epi) comprising *EPCAM*, *KRT19*, *KRT8*, and *KRT18*, and a phyllodes-like signature (S_Phy) comprising *CD34*, *ALDH1A1*, *HOXB13*, *WNT5A*, *LEF1*, *AXIN2*, and *CTNNB1*. Cells were classified as double-positive if both signature scores exceeded the 75th percentile threshold, a commonly used cutoff for identifying highly expressing subpopulations. Pearson correlation coefficients between signature scores were calculated using the SciPy library (version 1.9.0) to assess the independence of the epithelial and phyllodes-like transcriptional programs. Specifically, we employed an upper quartile (75th percentile) thresholding approach. This distribution-based, non-parametric method is widely utilized in single-cell transcriptomics to robustly identify high-expressing cell populations while accounting for the inherent sparsity and heterogeneity of gene expression data.

### 4.7. Statistical Analysis

Descriptive statistics were used to summarize patient demographics, clinical characteristics, and genomic alterations. Categorical variables were compared using chi-square or Fisher’s exact test, and continuous variables were compared using Student’s *t*-test or Mann–Whitney U test as appropriate. *p*-values less than 0.05 were considered nominally significant. Because of the rarity of the disease and the limited sample size, these analyses were exploratory in nature, and no adjustments for multiple comparisons were applied. All clinical and genomic statistical analyses were conducted using Microsoft Excel 2021 (Microsoft Corporation, Redmond, WA, USA) and Statcel 5 (OMS Publishing Inc., Saitama, Japan).

### 4.8. Use of Artificial Intelligence

The authors used AI-based tools (ChatGPT, Gemini, Grammarly, DeepL, Google Translation, and Claude) to improve the readability and linguistic precision of the manuscript, to assist in developing Python scripts, and to support the creation of the Graphical Abstract.

## Figures and Tables

**Table 1:** Clinical background of breast cancer cases in the C-CAT database.

Breast Cancer Cases (*n* = 8409)			
Age Group (years)		Representative Gene Alterations	
50–59	2724 (32.4%)	*TP53*	4455 (53.0%)
60–69	2159 (25.8%)	*PIK3CA*	3399 (40.4%)
40–49	1602 (19.1%)	*GATA3*	1674 (19.9%)
70–79	1296 (15.4%)	*BRCA2*	1624 (19.3%)
30–39	427 (5.1%)	*RAD21*	1597 (19.0%)
80–89	138 (0.2%)	*MYC*	1563 (18.6%)
20–29	53 (0.1%)	*ESR1*	1509 (17.9%)
10–19	5 (0.0%)	*NOTCH3*	1477 (17.6%)
90-	5 (0.0%)	*CCND1*	1408 (16.7%)
		*ERBB2*	1368 (16.3%)
Sex			
Male	51 (0.6%)	Cancer Genomics Panel	
Female	8358 (99.4%)	FoundationOne CDx	5930 (70.5%)
		GenMine^®^ TOP Cancer Panel	220 (2.6%)
		FoundationOne Liquid CDx	1516 (18.0%)
		Guardant360	125 (1.5%)
		NCC Oncopanel System	618 (7.3%)

The study period for each genome profile testing was as follows: NCC Oncopanel System (1 June 2019 to 13 August 2025), FoundationOne^®^ CDx (1 June 2019 to 18 August 2025), FoundationOne^®^ Liquid CDx (1 August 2021 to 18 August 2025), Guardant360^®^ CDx (24 July 2023 to 18 August 2025), and GenMine^®^ TOP Cancer Panel (1 August 2023 to 18 August 2025).

**Table 2:** Background of Metaplastic Cancer Cases Registered in the Center for Cancer Genomics and Advanced Therapeutics.

Total Metaplastic Carcinoma Cases (*n* = 123)			
Age Group (years; Median 56)		Cancer Genomics Panel	
50–59	42 (34.1%)	FoundationOne CDx	105 (85.4%)
60–69	23 (18.7%)	GenMine^®^ TOP Cancer Panel	7 (5.7%)
70–79	23 (18.7%)	FoundationOne Liquid CDx	5 (4.1%)
40–49	22 (17.8%)	Guardant360	2 (1.6%)
30–39	9 (7.3%)	NCC Oncopanel System	4 (2.4%)
20–29	3 (2.4%)		
80–89	1 (0.8%)	Estrogen Receptor Status	
		Positive	15 (12.2%)
Smoking History		Negative	99 (80.5%)
No	90 (73.2%)	Unknown	9 (7.3%)
Yes	22 (17.9%)		
Unknown	11 (8.9%)	Progesterone Receptor Status	
		Positive	9 (7.3%)
Drinking History		Negative	107 (87.0%)
No	106 (86.2%)	Unknown	9 (7.3%)
Yes	5 (4.1%)		
Unknown	12 (9.8%)	HER2 Status	
		0	73 (59.3%)
Family History (Cancer)		1+	28 (22.8%)
Yes	86 (70.0%)	2+ ISH negative/unknown	6 (4.9%)
No	20 (16.3%)	2+ ISH positive	2 (0.2%)
Unknown	17 (13.8%)	3+	5 (4.1%)
		unknown	9 (7.4%)
Germline *BRCA* status			
Positive	1 (0.8%)	Sample Specimen	
Negative	106 (86.2%)	Primary Site	77 (62.6%)
Unknown	16 (13.0%)	Metastatic Site	39 (31.7%)
		Blood	6 (4.9%)
Sex		Unknown	1 (0.8%)
Male	1 (0.8%)		
Female	122 (99.2%)	Subtypes	
		Spindle Cell Type	17 (13.8%)
		Squamous Cell Type	7 (5.7%)
		Chondroid Metaplasia	4 (3.3%)
		Osseous Metaplasia	3 (2.4%)
		Adenosquamous Type	2 (1.6%)
		Metaplastic Carcinosarcoma	1 (0.8%)
		Not Other Specified	89 (72.4%)

The study period for each genome profile testing was as follows: NCC Oncopanel System (1 June 2019 to 13 August 2025), FoundationOne^®^ CDx (1 June 2019 to 18 August 2025), FoundationOne^®^ Liquid CDx (1 August 2021 to 18 August 2025), Guardant360^®^ CDx (24 July 2023 to 18 August 2025), and GenMine^®^ TOP Cancer Panel (1 August 2023 to 18 August 2025). HER2: human Epithelial Growth Factor Receptor 2, ISH: in situ Hybridization.

**Table 3:** Cancer Genomic Profiling Tests Used in This Study (Reimbursed In Japan).

Features	FoundationOne CDx	FoundationOne Liquid CDx	NCC Oncopanel System	GenMine TOP Cancer Panel	Guardant 360
Sample Type	FFPE tissue	Blood	FFPE tissue	FFPE tissue	Blood
Number of Genes	324	324	114	723	73
MSI Testing	Yes	Yes	Yes *	No	Yes
TMB Assessment	Yes	Yes	Yes	Yes	No
Minimum Tumor Content Required	20%	N/A	20%	20%	N/A
Required DNA Input	50 ng	2 tubes	50 ng	50 ng	2 tubes

*: Earlier versions did not include microsatellite instability testing capability. FFPE: Formalin-Fixed Paraffin-Embedded, MSI: Microsatellite Instability, TMB: Tumor Mutation Burden, N/A: Not Applicable.

**Table 4:** Results of Metaplastic Cancer Genomic Testing Cases and Treatment Resistance registered in the Center for Cancer Genomics and Advanced Therapeutics.

Total Metaplastic Carcinoma Cases (*n* = 123)				
Druggable Variants: Molecular Board Review		Taxane Resistance-Associated Gene Alterations		
No	60 (48.8%)	Gene	OR	*p*-value
Yes	46 (37.4%)	*PIK3CA*	0.35	0.03
Unknown	17 (13.8%)	*CDKN2A*	1.30	0.59
		*TP53*	2.42	0.09
Microsatellite Instability		*PTEN*	1.31	0.60
Microsatellite Stable	104 (84.6%)			
Microsatellite Equivalent	1 (0.8%)	Anthracycline Resistance-associated Gene Alterations		
Unknown	18 (14.6%)	Gene	OR	*p*-value
		*PIK3CA*	0.69	0.51
Presumed Germline Pathogenic Variant		*CDKN2A*	1.13	0.85
No	119 (96.8%)	*TP53*	1.20	0.79
Yes	4 (3.3%)	*PTEN*	1.91	0.25

## Data Availability

The dataset generated during this study is not publicly accessible due to confidentiality agreements as part of the ethics approval process.

## Abbreviations

The following abbreviations are used in this manuscript:

MpBC	metaplastic breast carcinoma
CGP	comprehensive genomic profiling
C-CAT	Center for Cancer Genomics and Advanced Therapeutics
IHC	immunohistochemistry
ISH	in situ hybridization
TMB	tumor mutational burden
MSI	microsatellite instability
MSS	microsatellite stable
DCR	disease control rate
ORR	overall response rate
ER	estrogen receptor
PgR	progesterone receptor
HER2	human epidermal growth factor receptor 2
